# Extrathyroidal Extension Prediction of Papillary Thyroid Cancer With Computed Tomography Based Radiomics Nomogram: A Multicenter Study

**DOI:** 10.3389/fendo.2022.874396

**Published:** 2022-06-01

**Authors:** Pengyi Yu, Xinxin Wu, Jingjing Li, Ning Mao, Haicheng Zhang, Guibin Zheng, Xiao Han, Luchao Dong, Kaili Che, Qinglin Wang, Guan Li, Yakui Mou, Xicheng Song

**Affiliations:** ^1^ Department of Otorhinolaryngology, Head and Neck Surgery, Yantai Yuhuangding Hospital, Qingdao University, Yantai, China; ^2^ Shandong Provincial Clinical Research Center for Otorhinolaryngologic Diseases, Yantai, China; ^3^ Big data and Artificial Intelligence Laboratory, Yantai Yuhuangding Hospital, Qingdao University, Yantai, China; ^4^ Department of Radiology, Yantai Yuhuangding Hospital, Qingdao University, Yantai, China; ^5^ Department of Thyroid Surgery, Yantai Yuhuangding Hospital, Qingdao University, Yantai, China; ^6^ Second Clinical Medicine College, Binzhou Medical University, Yantai, Shandong, China

**Keywords:** papillary thyroid cancer, extrathyroidal extension (ETE), radiomics, contrast-enhanced CT, nomogram

## Abstract

**Objectives:**

To develop and validate a Computed Tomography (CT) based radiomics nomogram for preoperative predicting of extrathyroidal extension (ETE) in papillary thyroid cancer (PTC) patients

**Methods:**

A total of 153 patients were randomly assigned to training and internal test sets (7:3). 46 patients were recruited to serve as an external test set. A radiologist with 8 years of experience segmented the images. Radiomics features were extracted from each image and Delta-radiomics features were calculated. Features were selected by using one way analysis of variance and the least absolute shrinkage and selection operator in the training set. K-nearest neighbor, logistic regression, decision tree, linear-support vector machine (linear -SVM), gaussian-SVM, and polynomial-SVM were used to build 6 radiomics models. Next, a radiomics signature score (Rad-score) was constructed by using the linear combination of selected features weighted by their corresponding coefficients. Finally, a nomogram was constructed combining the clinical risk factors with Rad-scores. Receiver operating characteristic (ROC) curve, decision curve analysis (DCA), and calibration curve were performed on the three sets to evaluate the nomogram’s performance.

**Results:**

4 radiomics features were selected. The six models showed the certain value of radiomics, with area under the curves (AUCs) from 0.642 to 0.701. The nomogram combining the Rad-score and clinical risk factors (radiologists’ interpretation) showed good performance (internal test set: AUC 0.750; external test set: AUC 0.797). Calibration curve and DCA demonstrated good performance of the nomogram.

**Conclusion:**

Our radiomics nomogram incorporating the radiomics and radiologists’ interpretation has utility in the identification of ETE in PTC patients.

## Highlights

- Radiomics features were extracted from non-contrast and contrast-enhanced CT images, and Delta-radiomics features were calculated.
- 6 classifiers (KNN, LR, DT, linear-SVM, gaussian-SVM, and polynomial-SVM) were used to construct radiomics models and showed favorable discriminatory abilities.
- The nomogram model combining the radiomics and radiologists’ interpretation showed good predictive performance.

## Introduction

While thyroid cancer is one of the most common cancers worldwide, it has a very low mortality rate (1). Papillary thyroid cancer (PTC) is the most common histologic subtype of thyroid cancer (2, 3). In the 8^th^ edition of AJCC (American Joint Committee on Cancer) (4), extrathyroidal extension (ETE), which was subdivided into gross ETE and microscopic ETE, refers to the primary tumor invades the surrounding structures including the strap muscles, trachea, vasculature, larynx, esophagus, and recurrent laryngeal nerve. Furthermore, ETE is an independent risk factor associated with lymph node metastasis and directly affects the clinical outcomes of PTC patients (5, 6). The 15-year survival rate of PTC patients with ETE is significantly lower than that of patients without ETE. Therefore, the diagnosis of ETE is essential for the treatment decision of PTC.

Ultrasound (US), magnetic resonance imaging (MRI), and Computed Tomography (CT) are common non-invasive imaging modality for preoperative ETE diagnosis. US has a high degree of accuracy and sensitivity for identifying ETE (~ 70%), but low specificity (27.2%-68.9%) leads to a high risk of false positive results (7, 8), and US relying on the experience level of the operator is subjective. MRI is slightly inferior to US but similar to CT with regards to accuracy (9). Young Lan Seo et al. (10), found that CT could accurately identify most types of ETE (i.e., esophageal invasion). Furthermore, contrast-enhanced computed tomography (CE-CT) offers an additional phase (venous phase) and three-dimensional images. However, it is difficult to accurately predict ETE based only on traditional images, and obtain the guidance needed for preoperative planning, because interpretation of the images depends on the experience level of the radiologist or surgeon. Besides, histopathological examination, which is the gold standard for diagnosing ETE, is an invasive method, and usually used to verify a preoperative assessment of ETE obtained by using non-invasive tools. Thus, a novel non-invasive tool is needed to help surgeons predict ETE and make clinical decisions prior to performing an operation.

In recent years, Radiomics, the high-throughput extraction of large amounts of image features from radiographic images, can be used to build a mineable high-dimensional database of original material obtained by machine learning (11). Radiomics can also be used to identify the underlying heterogeneity of images that can be difficult to detect with human eyes. Combined with Machine leaning, radiomics are generally used by researchers to predict the prognosis of patients with tumors and assess the impact of various diseases (12, 13). Until now, no study has built a predictive model of ETE using a CT radiomics nomogram adding to time sequential features and then validated the model in a multicenter study. We have achieved that goal.

In this study, we established an effective radiomics nomogram capable of predicting the ETE of PTC based on CT images.

## Methods

### Patients

**Figure 1:**
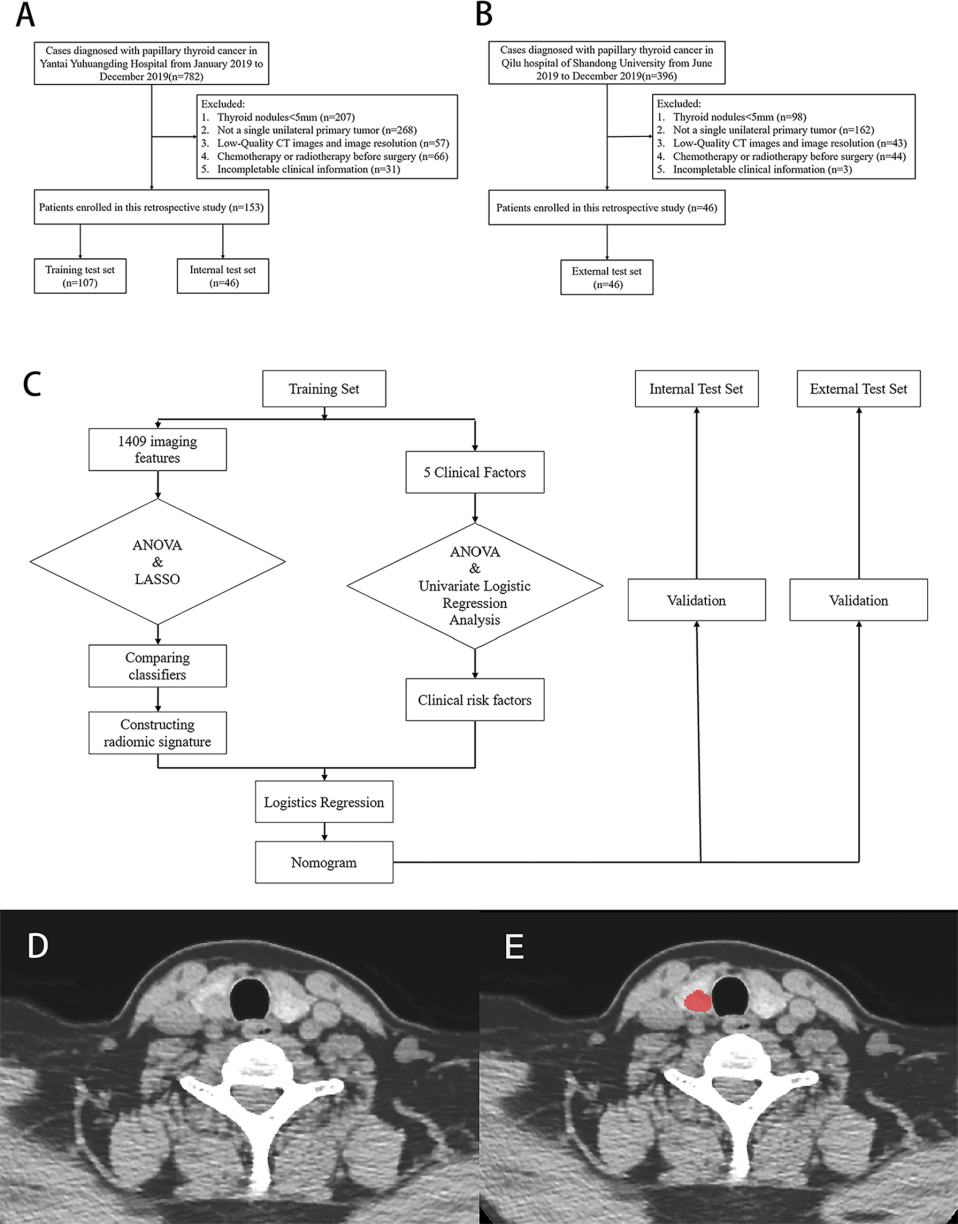
Flow chart of patients’ enrolment in **(A)** training, internal test set and **(B)** external test set. **(C)** Flow chart of study work. **(D, E)** Examples of regions of interest (ROIs) segmentation on contrast-enhanced computed tomography (CE-CT) images.

This was a retrospective, multicenter, and diagnostic study. From January 2019 to December 2019, 153 patients, which were collected consecutively from Yantai Yuhuangding Hospital, were randomly assigned to a training set or an internal test set at a ratio of 7:3 (**Figure 1A**). Additionally, 46 consecutive patients at Qilu Hospital of Shandong University were recruited to serve as an external test set from June 2019 to December 2019 (**Figure 1B**). The inclusion and exclusion criteria can be found in **Appendices**.

The protocol for this retrospective study was approved by the Institutional Review Board of The Affiliated Yantai Yuhuangding Hospital of Qingdao University. The requirement for obtaining written informed consent from the patients was waived by the review board. A study work flow diagram is shown in **Figure 1C**.

### Radiologists’ Interpretation of ETE

CT assessments of ETE were performed by two experienced radiologists, one with 15 years of experience and another with 5 years of experience. Both radiologists were blinded to histopathological results. Any disagreement was resolved by discussion or consultation with a third radiologist who had 20 years of experience. ETE was reported on CT images when at least one of the following CT criteria was fulfilled: 1) tumor in contact with 180° or more of the tracheal, esophageal, muscle or vascular circumference; 2) loss of normal structure; 3) a clinical symptom (e.g., ipsilateral vocal cord palsy) was present that could be explained by the CT images; 4) tumor showed poorly defined margin with heterogeneous signal intensity in adjacent soft tissue; 5) focal bulging out or disruption of the thyroid capsule by tumor; 6) more than 25% of perimeter of the tumor was abutting the thyroid capsule (10). Kendall’s coefficient of concordance *W* (Kendall’s *W*) was used to evaluate inter-radiologist agreement. *W* scores range between 0 and 1, which was graded as very good (0.80 to 1.00), good (0.60 to 0.80), fair (0.40 to 0.60), moderate (0.20 to 0.40) or poor (<0.20) (14).

### Image Segmentation

The Image Acquisition protocol is shown in **Appendices**. After that, a radiologist with 8 years of experience manually delineated the PTC regions of interest (ROI) (15) on non-contrast and venous phase CT images by using an ITK-SNAP (version 3.8.0; www.itksnap.org). Based on their traits, ROIs were manually delineated along the tumor contour on each transverse section and the ROIs in each slice constituted a volume of interest (VOI). A sample of segmentation process is shown in **Figures 1D, E**. Three months later, another two radiologists segmented the images of 30 patients who were randomly selected to assess the intra- and inter-observer reproducibility of radiomics features by intra- and inter-class correlation coefficients (ICCs). An ICC > 0.8 indicated an excellent agreement of radiomics features.

### Feature Extraction

Before feature extraction, VOIs were prepossessed included gray value standardization, gray level discretization, and image resampling. A total of 2818 radiomics features were originally extracted from each VOI by using PyRadiomics (16) on Python (version 3.7). A total of 1409 sequential features based on time series were calculated as Delta _(radiomics feature)_, which was defined as the difference between features seen on the venous phase and non-contrast CT images. Delta _(radiomics feature)_ was calculated as follows:


$${\text{Delta}}_{\left(\text{radiomics feature}\right)}={\text{Feature}}_{\left(\text{venous phase}\right)}-{\text{Feature}}_{\left(\text{non-contrast phase}\right)}$$

A total of 4227 features (containing radiomics features and sequential features) were identified for each patient containing 19 types of first order statistics, 13 types of Shape features, 28 types of Gray-Level Co-occurrence Matrix (GLCM) features, 16 types of Gray-Level Run-Length Matrix (GLRLM) features, 16 types of Gray Level SizeZone Matrix (GLSZM) features, and 56 types of sequential features.

### Features Selection

The values of the extracted features were standardized with z scores by using mean and standard deviation values. Next a two-step procedure for dimensionality reduction and feature selection was devised. First, an analysis of variance (ANOVA) was performed to screen out discriminative features in the training set, with only features having a *P* < 0.05 being selected. Next, the least absolute shrinkage and selection operator (LASSO) method was used to reduce the dimensions of features, identify the most significant features (17), and make a second selection.

### Radiomics Models Construction and Evaluation

K-Nearest Neighbor (KNN), Logistics Regression (LR), Decision Tree (DT), Linear-support vector machine (Linear-SVM), Gaussian-SVM, and Polynomial-SVM methods were used to construct radiomics models, respectively. Area under the curve (AUC) was regarded as a performance indicator and used to evaluate the performance of the radiomics-based models built by using each classifier. Finally, a radiomics signature (Rad-score) was calculated by using a linear combination of selected features weighted by the corresponding LASSO coefficients.

### Clinical Risk Factors Selection and Nomogram Construction

In the training set, one-way ANOVA and multivariate logistic regression analysis were used to screen clinical risk factors including sex, age, primary site, tumor diameter, and radiologist interpretation to identify the clinical risk factors for ETE. To quantify the roles of the Rad-score and clinical risk factors in predicting ETE, both of those risk factors were included in a multivariate logistic regression analysis that was conducted using a backward-stepwise approach, where collinearity was considered and risk factors with a variance inflation factor (VIF) > 10 and a *P* > 0.05 were excluded. When the minimum Akaike information criterion was reached, the Akaike information was taken as the criterion, the stepwise procedure was stopped, and the final multivariate logistic regression constituted the nomogram.

### Nomogram Validation

The nomogram’s predictive performance was evaluated in the training set, internal test set, and external test set, respectively. The nomogram’s performance was evaluated in terms of the receiver operating characteristic (ROC) curve. In particular, the comparison between the nomogram and radiologists’ interpretation was illustrated in the same ROC curve. A decision curve analysis (DCA) was plotted for the entire set and applied in a clinical usefulness evaluation of the nomogram by calculating the net benefits at different threshold values in the training and test sets. In addition, a calibration (agreement between observations and prediction of ETE) curve was used to evaluate the agreement between actual status and ETE probabilities as predicted by the nomogram, accompanied by the Hosmer-Lemeshow test.

### Statistical Analysis

All statistical analyses were performed using Python (version 3.6, https://www.python.org), R software (version 4.0.3, https://www.r-project.org), and SPSS (version 26.0, IBM Corp.). Scikit-Learn, a Python library, was used for selecting radiomics features and constructing a radiomics model. The modules of “feature-selection,” “linear-model,” “svm,” “neighbors,” “tree,” and “metrics” were used for the entire procedure. The “rms” package in R software was used to select clinical risk factors, build the nomogram, and plot calibration curves. The “rmda” package was used to perform DCA. SPSS software was used to compare categorical variables (i.e., sex, T stage, primary site, radiologists’ interpretation, and lymph node metastasis) by using the χ^2^ test or Fisher’s exact test. Continuous variables were compared by using the student’s t test or Mann-Whitney U test, when appropriate. Statistical significance was two-sided, and a P < 0.05 was considered to be statistically significant.

## Results

### Clinical Characteristics

The numbers of enrolled patients in the training set, internal test set, and external test set were 107, 46, and 46, respectively. There were no significant differences in sex, age, diameter, Thyroid Stimulating Hormone (TSH), Free Thyroxine (FT4), Free Triiodothyronine (FT3), and Primary site in each data set, but there was a significant difference in T stage. Details of clinical characteristics in the 3 sets are shown in **Table 1**. Radiologist A and radiologist B reported all CT imaging results with good inter-radiologist agreement (Kendall’s W, 0.823).

**Table 1:** Characteristic of enrolled patients in three sets.

		Training Set (n = 107)			Internal Test Set (n = 46)			External Test Set (n = 46)		
		ETE (n = 28)	Non-ETE (n = 79)	*P* value	ETE (n = 12)	Non-ETE (n = 34)	*P* value	ETE (n = 13)	Non-ETE (n = 33)	*P* value
Sex (M/F)		5/23	27/52	0.105	1/11	6/28	0.440	2/11	11/22	0.223
Age (Years)^a^		44.82 ± 12.41	47.14 ± 12.07	0.388	53.42 ± 13.06	48.88 ± 13.36	0.315	40.77 ± 14.66	39.39 ± 12.17	0.764
Diameter (mm)^a^		1.53 ± 0.81	1.41 ± 1.29	0.627	1.28 ± 0.65	1.86 ± 1.35	0.059	1.77 ± 0.61	1.78 ± 1.12	0.962
Hormone (mIU/L)^a^	TSH	2.53 ± 1.03	2.14 ± 1.31	0.480	4.43 ± 7.71	2.20 ± 1.57	0.362	123.92 ± 181.05	54.44 ± 84.77	0.30
	FT4	15.91 ± 3.02	16.01 ± 2.27	0.861	15.50 ± 1.90	16.24 ± 2.60	0.390	2.77 ± 1.94	2.26 ± 1.36	0.328
	FT3	4.84 ± 0.84	4.82 ± 0.61	0.936	5.08 ± 1.47	4.85 ± 0.74	0.504	11.95 ± 2.08	13.85 ± 2.06	0.01*
T Stage	T_1_	6 (21.43)	61 (53.16)	<0.001*	4 (33.33)	22 (64.71)	0.014*	3 (23.08)	26 (78.78)	<0.001*
	T_2_	1 (3.57)	14 (1.27)		1 (8.33)	9 (26.47)		0(0.00)	5 (15.15)	
	T_3_	15 (53.57)	4 (45.57)		5 (41.67)	3 (8.82)		5 (38.46)	2 (6.06)	
	T_4_	6 (21.43)	0 (0.00)		2 (16.67)	0 (0.00)		5 (38.46)	0 (0.00)	
Primary site	Left/right lobe	28 (100.00)	77 (97.47)	0.395	12 (100.00)	33 (97.06)	0.548	13 (100.00)	32 (96.97)	>0.999
	Isthmus	0 (0.00)	2 (2.53)		0 (0.00)	1 (2.94)		0 (0.00)	1 (3.03)	
Radiologists’ Interpretation	correct	21 (75.00)	64 (81.01)	0.449	7 (58.33)	29 (85.29)	0.052	10 (76.92)	23 (69.70)	0.729
	incorrect	7 (25.00)	15 (18.99)		5 (41.67)	5 (14.71)		3 (23.08)	10 (30.30)	
LN Status	positive	19 (67.86)	32 (40.51)	0.013*	8(66.67)	10 (29.41)	0.157	10 (76.92)	18 (54.55)	0.197
	negative	9 (32.14)	47 (59.49)		4 (33.33)	24 (70.59)		3 (23.08)	15(45.45)	

The data are displayed as n (%) except otherwise noted.

^a^Mean ± standard deviation.

ETE, Extrathyroidal Extension; M, Male; F, Female; TSH, Thyroid Stimulating Hormone; FT4, Free Thyroxine; FT3, Free Triiodothyronine; LN, lymph node.

*P < 0.05

### Radiomics Features and Prediction Performance of the Radiomics-Based Models

**Figure 2:**
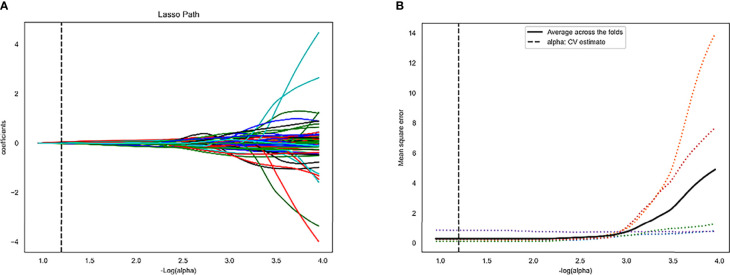
Least absolute shrinkage and selection operator (LASSO) algorithm for radiomics features selection. **(A)** LASSO coefficient profiles of the 4 features. The y-axis represents coefficient of each feature. **(B)** Mean square error path.

A total of 4227 radiomics features were extracted from each patient’s images. The intra-observer and inter-observer ICCs were 0.867 and 0.915, respectively, indicating an excellent agreement on radiomics features. Four radiomics features (Small Area Low Gray Level Emphasis, Gray Level Variance, Difference Entropy, and Busyness) in the training set were finally selected (**Figure 2**; **Table 2**).

**Table 2:** LASSO coefficient profiles of the 4 features.

Radiomics features	Phase	Feature type	Coefficient
Small Area Low Gray Level Emphasis	Non-contrast	GLSZM	0.0426553
Gray Level Variance	Non-contrast	GLSZM	-0.023849
Difference Entropy	Non-contrast	GLSZM	0.014683
Busyness	Non-contrast	GLSZM	-0.007175

LASSO, least absolute shrinkage and selection operator; GLSZM, gray-level size zone matrix.

**Figure 3:**
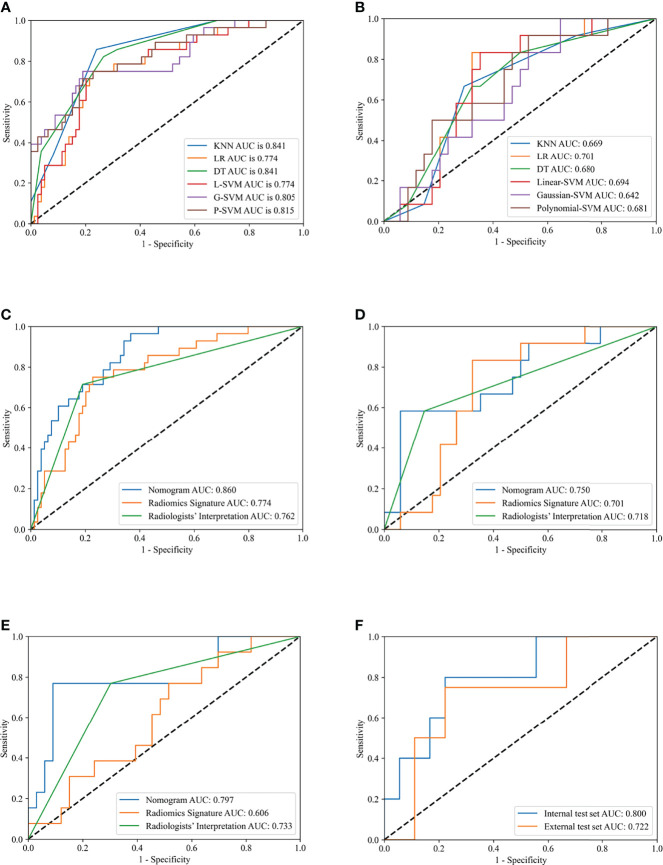
Receiver operating characteristic (ROC) curves of different models in training and test sets. Receiver operating characteristic (ROC) curves of K-Nearest Neighbor (KNN), Logistics Regression (LR), Decision Tree (DT), Linear-support vector machine (Linear-SVM), Gaussian-SVM, and Polynomial-SVM models in the training **(A)** and internal test **(B)** set. ROC curves of nomogram, radiomics signature, and radiologists’ interpretation in the training **(C)**, internal test **(D)**, external test **(E)** set. ROC curves of nomogram for division of minimal and gross extrathyroidal extension (ETE) in the internal test and external test set **(F)**.

Predictive models built by using KNN, LR, DT, Linear-SVM, Gaussian-SVM, and Polynomial-SVM, showed favorable discriminatory abilities for radiomics parameters with AUCs of 0.841 (95% Confidence Interval [CI], 0.769-0.900), 0.774 (95% CI, 0.683-0.853), 0.841 (95% CI, 0.772-0.897), 0.774 (95% CI, 0.680-0.854), 0.805 (95% CI, 0.710-0.879), and 0.815 (0.708-0.888), respectively, in the training set (Figure 3 A). In the internal test set, the six radiomics models also showed good performance with AUCs of 0.669 (95% Confidence Interval [CI], 0.394-0.568), 0.701 (95% CI, 0.480-0.767), 0.680 (95% CI, 0.516-0.795), 0.694 (95% CI, 0.508-0.788), 0.642 (95% CI, 0.454-0.600), and 0.681 (0.466-0.700), respectively (**Figure 3B**). Interestingly, in terms of AUC, the LR, which was later used to build the nomogram, demonstrated the best performance of the 6 models.

### Development and Validation of the Radiomics Nomogram

The 4 features mentioned above were selected and their coefficients were plugged into a formula for calculating Rad-score as described below:


Rad−score=Small Area Low Gray Level Emphasis∗0.0426553682266122+Gray Level Variance∗−0.0238497391861649+Difference Entropy∗0.0146833132887633+Busyness∗−0.00717595762541243

**Figure 4:**
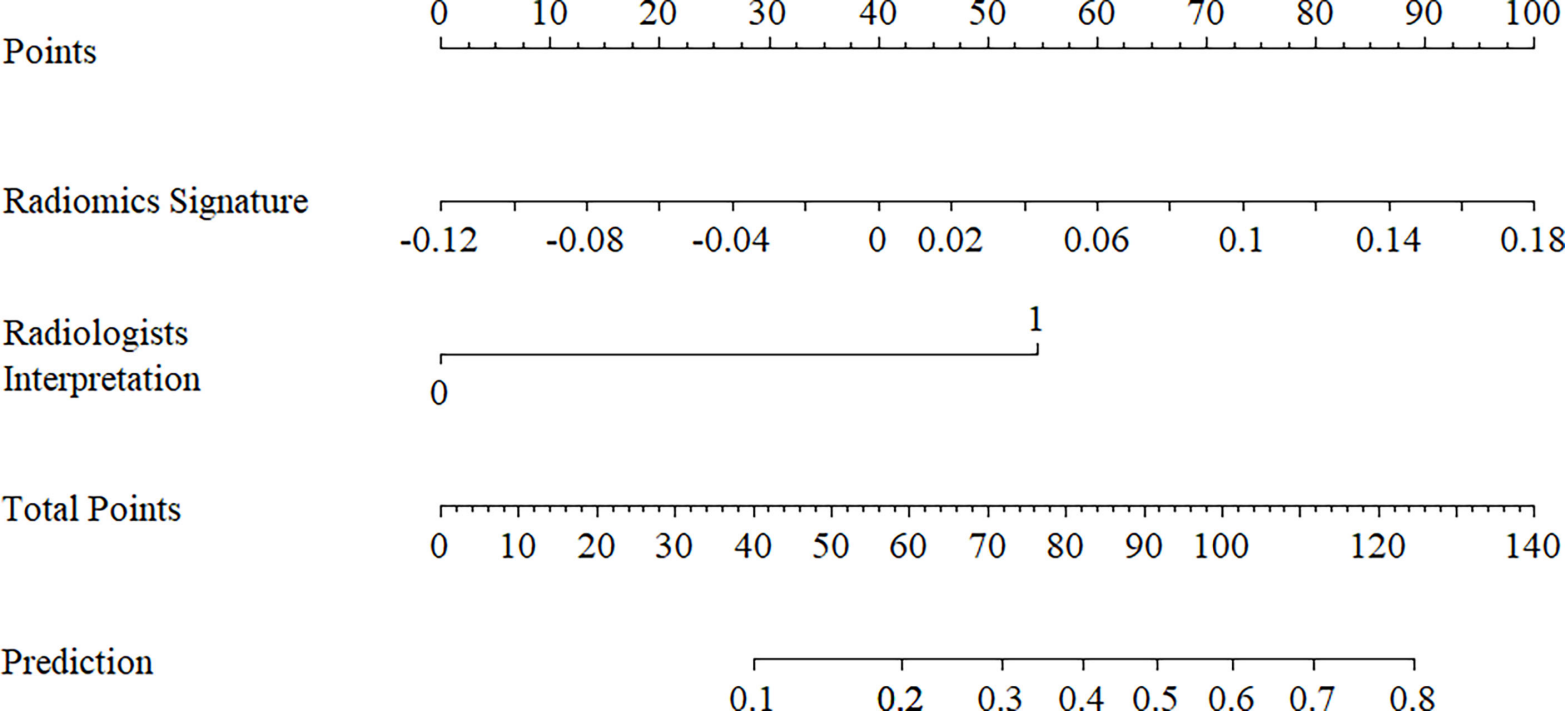
Radiomics nomogram with radiomics signature (rad-score) and radiologists’ interpretation incorporated.

**Figure 5:**
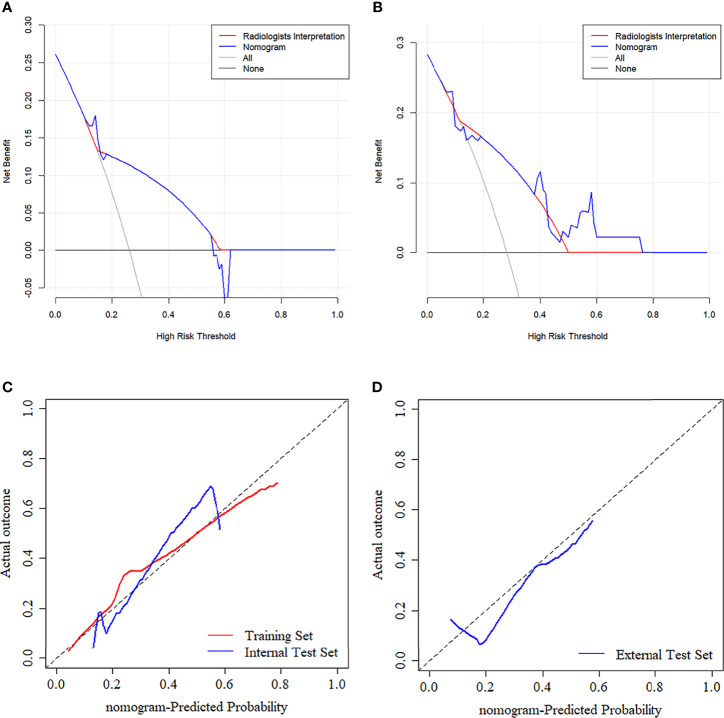
**(A, B)** Decision curve analysis (DCA) for the prediction models in the internal and external test set. The y-axis represents the net benefits, while the x-axis represents the threshold probability. The blue line represents the radiomics nomogram. The red line represents the radiologists’ interpretation model. The gray line represents the assumption that no patients were diagnosed as ETE. The horizontal black line represents the assumption that all patients were diagnosed as ETE. Calibration curves of radiomics nomogram in the training **(C)**, internal test **(C)**, and external test **(D)** sets. The diagonal line represents the perfect prediction of the radiomics nomogram.

The ANOVA and multivariate logistic regression analysis only identified radiologists’ interpretation as being an independent ETE predictor (**Table 3**). The values of Rad-score and radiologists’ interpretation for predicting the presence of ETE in PTC patients were quantified in our nomogram (score for each factor is shown in **Figure 4**). The radiomics nomogram showed a good prognostic capability, with AUCs of 0.860 (95% CI, 0.790-0.931), 0.750 (95% CI, 0.579-0.921), and 0.797 (95% CI, 0.665-0.926) in the training, internal test, and external test sets, respectively (**Figures 3C–E**; **Table 4**). Furthermore, the radiomics nomogram had a more powerful predictive efficiency than the interpretation of radiologists with 5 and 10 years of experience, whose AUC in the training, internal test, and external test sets were 0.762 (95% CI, 0.667-0.858), 0.718 (95% CI, 0.560-0.876), and 0.733 (95% CI, 0.615-0.841), respectively (**Figures 3C–E**). In the external test set, the nomogram’s accuracy (0.848 vs. 0.717) and specificity (0.909 vs, 0.697) were higher than those of radiologist’s interpretation, reflecting a better prognostic performance of the radiomics nomogram; however, the sensitivity of the nomogram (0.692, 95% CI 0.389-0.896) was similar with that of radiologists’ interpretation (0.769, 95% CI 0.460-0.938) (**Table 4**). We tested this nomogram to distinguish minimal and grossly ETE in internal and external test set, and achieved relatively good performance with AUC of 0.800 (0.578-1.000) and 0.722(0.390-1.000), sensitivity of 0.800 (0.299-0.989) and 0.500(0.092-0.908), specificity of 0.667 (0.412-0.856) and 0.889(0.507-0.994), and accuracy of 0.696 (0.471-0.868) and 0.769(0.462-0.950) respectively. (**Figure 3F**). The DCA showed that radiomics nomogram could add more benefit than treat patients as all-ETE or non-ETE when the threshold probability ranged from 0.10 to 0.50 in internal test set and 0.12 to 0.75 in the external test set (**Figures 5A, B**). The calibration curve for the radiomics nomogram, when assessed by the Hosmer-Lemeshow test, yielded *P* of 0.48, 0.88, and 0.34 in the training set, internal test set, and external test set, respectively (**Figures 5C, D**), demonstrated good agreement between actual status and ETE probabilities as predicted by the nomogram in all three sets.

**Table 3:** The selection of clinical risk factors for ETE by ANOVA and univariate LR analysis.

Variables	ANOVA		Univariate LR Analysis	
	F Value	P	OR	P
Sex	2.642	0.107	NA	NA
Age	0.752	0.388	NA	NA
Primary Site	0.612	0.436	NA	NA
Tumor Diameter	0.065	0.799	NA	NA
Radiologist’s Interpretation	33.412	<0.01*	1.506	<0.01*

ETE, extrathyroidal extension; ANOVA, analysis of variance; LR, logistic regression; OR, odds ratio; NA, not available.

*P < 0.05

**Table 4:** Efficacies of the models predicting ETE in patients with PTC.

	AUC	Sensitivity	Specificity	Accuracy
**Training Set**				
Rad-score	0.774 (0.677-0.871)	0.722 (0.671-0.861)	0.750 (0.571-0.893)	0.757 (0.665-0.835)
Radiologists’ interpretation	0.762 (0.667-0.858)	0.810 (0.712-0.886)	0.714 (0.536-0.857)	0.785 (0.695-0.859)
Nomogram	0.860 (0.790-0.931)	0.734 (0.633-0.853)	0.786 (0.643-0.929)	0.710 (0.615-0.794)
**Internal Test Set**				
Rad-score	0.701 (0.545-0.857)	0.706 (0.588-0.882)	0.583 (0.333-0.833)	0.674 (0.520-0.805)
Radiologists’ interpretation	0.718 (0.560-0.876)	0.853 (0.706-0.971)	0.583 (0.333-0.833)	0.783 (0.636-0.891)
Nomogram	0.750 (0.579-0.921)	0.765 (0.584-0.886)	0.583 (0.333-0.833)	0.717 (0.565-0.840)
**External Test Set**				
Rad-score	0.606 (0.469-0.748)	0.692 (0.389-0.896)	0.485 (0.312-0.661)	0.543 (0.390-0.691)
Radiologists’ interpretation	0.733 (0.615-0.841)	0.769 (0.460-0.938)	0.697 (0.511-0.838)	0.717 (0.565-0.840)
Nomogram	0.797 (0.665-0.926)	0.692 (0.389-0.896)	0.909 (0.745-0.976)	0.848 (0.711-0.937)

ETE, extrathyroidal extension; PTC, Papillary thyroid cancer; Rad-score, radiomics signature score; AUC, area under curves.

## Discussion

Most patients with PTC have a favorable long-term survival prognosis (18); however, ETE remains an independent risk factor affecting overall survival and clinical decision making (19). Therefore, it is crucial to create a powerful predictive tool that can help surgeons and inexperienced radiologists evaluate possible ETE and make a clinical decision regarding treatment. In this retrospective multicenter study, six predictive models were established for verifying that radiomics could be used to predict ETE. Furthermore, a nomogram was constructed based on radiomics information and clinical risk factors, and displayed an excellent ability to predict ETE, with AUCs of 0.750 and 0.797 in an internal test set and external test set, respectively. Those results indicated that the radiomics nomogram could serve as an independent medical decision-making tool, and satisfy the requirements of precision medicine.

Unlike previous studies (20–24), the present study investigated the value of radiomics with six classifiers. This result showed that radiomics could play a crucial role in ETE prediction. Nomograms based on radiomics have been widely used to predict medical prognoses (25) and clinical outcomes by combining a Rad-score and clinical risk factors (26). The nomogram constructed in this study demonstrated an ability to generate individualized predictions that were useful for identifying and stratifying patients with PTC.

Some previous studies have been conducted on this topic (27, 28). Bin Chen et al. (27) and Xian Wang et al. (28) constructed radiomics nomograms for ETE prediction based on CT images and US images, respectively. Nevertheless, their studies lacked an external test set. Furthermore, those investigators only used a single classifier (logistic regression analysis) to evaluate the radiomics approach and build a nomogram. Their results showed that the nomogram had a discrimination ability (AUCs of 0.772 and 0.824, respectively, in an internal test set) for ETE classification that was similar to that of our nomogram (AUC = 0.750 in internal test set). When comparing the two nomograms, the nomogram developed in our multicenter study has notable advantages. First, the use of an external test set improved the reliability of our radiomics nomogram and proved that it had relatively good repeatability and generalization ability. This is the first study to use a CT radiomics nomogram adding to time sequential features which greatly enhanced a variety of features to predict ETE. However, no single feature was proven to be statistically significant, which means the time change of CT images may play a minor role in ETE prediction. Finally, the ability of this radiomics nomogram to predict ETE approximated that of experienced radiologists’ interpretation, showing that the nomogram could be used to help surgeons make clinical decisions prior to performing surgery, and satisfy the requirements of precision medicine.

In addition, 4 selected features were all from non-contrast CT images, suggesting that there was no obvious advantage of features extracted from CE-CT images in ETE diagnosis. However, CE-CT is useful in ETE interpretation.

The limitations of this study are as follows: (1) PTCs with a diameter < 1 cm were included in the study, and may have been difficult to distinguish on CT images; this may have led to sample bias. To address this problem, any doubt was resolved by discussion or consultation with a third radiologist who had 20 years of experience. (2) The study’s retrospective design may have led to selection bias. Prospective studies are needed to control for confounding variables. (3) A manual segmentation approach is time-consuming and may affect the precision of feature extraction in some cases; however, the ICCs for intra- and inter-observer agreement ranged from 0.809 to 0.923. Previous studies showed that the automatic segmentation method may facilitate the use of radiomics in busy clinical practice and lead to high degrees of intra- and inter-observer reproducibility (29–32). A further study will use automatic segmentation to draw the ROIs. (4) CE-CT leads to higher total radiation doses, longer examination times, and use of contrast medium, limiting its broad use in clinical practice. (5) Only the machine learning method was used, and previous studies have shown that deep learning methods have a certain value for predicting lymph node metastases and classifying PTC (33, 34); however, no study has ever constructed an ETE-prediction model by using deep learning methods. In light of this, we will focus on that in a subsequent study.

## Conclusion

In this study, a non-invasive nomogram combined the Rad-score and radiologists’ interpretation showing good repeatability and generalization ability for predicting ETE. This radiomics nomogram may also facilitate clinical decision-making; however, additional studies with larger sample sizes and more centers should be performed to improve the nomogram’s efficiency.

## Data Availability Statement

The raw data supporting the conclusions of this article will be made available by the authors, without undue reservation.

## Ethics Statement

The studies involving human participants were reviewed and approved by the Institutional Review Board of The Affiliated Yantai Yuhuangding Hospital of Qingdao University. Written informed consent for participation was not required for this study in accordance with the national legislation and the institutional requirements.

## Author Contributions

PY, XW, and JL implemented the literature searching and manuscript writing. KC, QW, and NM implemented the ETE interpretation. GL implemented ROI segmentation. HZ contributed to data analysis, figure making, and algorithm development. YM, GZ, XH, and LD identified the radiological characteristics of PTC and estimated and adjusted the accuracy of ROIs. XS, YM, and NM conducted the design, quality control, and data interpretation of this study. All authors analyzed the data and their significance and were involved in the final editing and approval of the submitted article. All authors contributed to the article and approved the submitted version.

## Funding

This work was supported by the Taishan Scholars Project (No. ts20190991).

## Conflict of Interest

Authors declared that the research was conducted in the absence of any commercial or financial relationships that could be construed as a potential conflict of interest.

## Publisher’s Note

All claims expressed in this article are solely those of the authors and do not necessarily represent those of their affiliated organizations, or those of the publisher, the editors and the reviewers. Any product that may be evaluated in this article, or claim that may be made by its manufacturer, is not guaranteed or endorsed by the publisher.

## Abbreviations

AJCC, American Joint Committee on Cancer; ANOVA, analysis of variance; AUC, area under the curve; CE-CT, contrast-Enhanced computed tomography; CI, confidence interval; CT, Computed Tomography; DCA, decision curve analysis; DT, decision Tree; ETE, extrathyroidal extension; ICCs, intra- and inter-class correlation coefficients; KNN, k-nearest neighbor; LASSO, least absolute shrinkage and selection operator; LR, logistic regression; MRI, magnetic resonance imaging; PTC, papillary thyroid cancer; ROC, Receiver Operating Characteristic; SVM, support vector machine; US, Ultrasound; VIF, variance inflation factor; VOI, volume of interest.
