# Supplementary material for: Extrathyroidal Extension Prediction of Papillary Thyroid Cancer With Computed Tomography Based Radiomics Nomogram: A Multicenter Study
Source: Front Endocrinol (Lausanne). 2022 Jun 1;13:874396. doi: 10.3389/fendo.2022.874396 (PMC9198261; doi:10.3389/fendo.2022.874396)
Supplement: Supplementary file 1 [file DataSheet_1.docx]

Supplementary Material

### Image Acquisition

All 153 patients underwent CE-CT that was performed with a 64-slice spiral CT scanner (SIEMENS, Germany) or 256-slice spiral CT scanner (Philips, Netherlands) at The Affiliated Yantai Yuhuangding Hospital of Qingdao University and Qilu Hospital of Shandong University prior to surgery. The details of the scan parameters are as follows: 120 kV; 300 mAs; section thickness, 2.5 mm; layer space, 2.5 mm; pitch, 0.97; image resolution, 512 x 512. All scans were performed in a craniocaudal direction from the level of the skull base to the subclavian level; this ensured that the entire thyroid was covered. At the start of each routine CT scan, the patient was administered 80-100 mL of iodopazone at a rate of 3.5-4 mL/s and given 30 mL of physiological saline. Next, CE-CT was performed after delays of 45 s (venous phase). The non-contrast scans and 1 contrast-enhanced CT images (venous phase) were exported in DICOM format from the picture archiving and communication system of our hospital, and imported into ITK-SNAP (version 3.8.0, www.itksnap.org).

### Inclusion and Exclusion Criteria

The study inclusion criteria were as follows: 1) a CE-CT examination that included non-contrast and venous phase CT images was performed within 2 weeks prior to surgery; 2) final histopathology confirmed the diagnosis of PTC with single unilateral lesion and provided the ETE or non-ETE result; 3) clinical data, except histopathological diagnosis, and CE-CT imaging data were available before surgery; 4) patients ≥ 18 years of age; 5) The thyroid nodules were ≥ 5 mm in diameter. The study exclusion criteria were as follows: 1) thyroid nodules that could not be segmented due to low-quality CT images (e.g., severe artifacts) or low image resolution; 2) patients who received any chemotherapy or radiotherapy for thyroid malignancy prior to surgery; 3) clinical information was incomplete. Clinical information (sex, age, primary site, tumor diameter, histopathological diagnosis, Free Triiodothyronine [FT3], Free Thyroxine [FT4], Thyroid Stimulating Hormone [TSH], T stage, and metastasis) for 153 enrolled patients was collected; all the patients had been hospitalized.
